# Butyrate to combat obesity and obesity‐associated metabolic disorders: Current status and future implications for therapeutic use

**DOI:** 10.1111/obr.13498

**Published:** 2022-07-20

**Authors:** Thirza van Deuren, Ellen E. Blaak, Emanuel E. Canfora

**Affiliations:** ^1^ Department of Human Biology, School for Nutrition and Translational Research in Metabolism (NUTRIM) Maastricht University Medical Center+ Maastricht The Netherlands

**Keywords:** butyrate, insulin resistance, microbiology, obesity

## Abstract

Evidence is increasing that disturbances in the gut microbiome may play a significant role in the etiology of obesity and type 2 diabetes. The short chain fatty acid butyrate, a major end product of the bacterial fermentation of indigestible carbohydrates, is reputed to have anti‐inflammatory properties and positive effects on body weight control and insulin sensitivity. However, whether butyrate has therapeutic potential for the treatment and prevention of obesity and obesity‐related complications remains to be elucidated. Overall, animal studies strongly indicate that butyrate administered via various routes (e.g., orally) positively affects adipose tissue metabolism and functioning, energy and substrate metabolism, systemic and tissue‐specific inflammation, and insulin sensitivity and body weight control. A limited number of human studies demonstrated interindividual differences in clinical effectiveness suggesting that outcomes may depend on the metabolic, microbial, and lifestyle‐related characteristics of the target population. Hence, despite abundant evidence from animal data, support of human data is urgently required for the implementation of evidence‐based oral and gut‐derived butyrate interventions. To increase the efficacy of butyrate‐focused interventions, future research should investigate which factors impact treatment outcomes including baseline gut microbial activity and functionality, thereby optimizing targeted‐interventions and identifying individuals that merit most from such interventions.

**Abbreviations**
acetyl‐CoA: acetyl coenzyme A
ATP: adenosine triphosphate
BAT: brown adipose tissue
BMI: body mass index
FFA: free fatty acid
FGF21: fibroblast growth factor 21
FMT: fecal microbial transplantation
GIP: glucose‐dependent insulinotropic polypeptide
GLP‐1: glucagon‐like peptide 1
GOS: galacto‐oligosaccharidesMCT‐1; monocarboxylate transporter 1
GPR: G protein‐coupled receptors
GPR41: G protein‐coupled receptor 41
GPR43: G protein‐coupled receptor 43
GPR109A: G protein‐coupled receptor 109A
HATs: histone acetylases
HbA_1c_
: glycated hemoglobin
HDACi: histone deacetylase inhibitors
HFD: high fat diet
HOMA‐IR: homeostatic model assessment for insulin resistance
MetS: metabolic syndrome
NAFLD: nonalcoholic fatty liver disease
PYY: peptide YY
RCT: randomized controlled trial
SCFA: short chain fatty acids
SMCT‐1: sodium‐coupled monocarboxylate transporter 1
T2DM: type 2 diabetes
WAT: white adipose tissue

## 1 INTRODUCTION

The prevalence of obesity has been on the rise for the last 50 years and is currently still rising at an alarming rate.^1^, ^2^ Evidence is accumulating that hints towards a relationship between the gut microbiome and the development of obesity and obesity‐associated complications such as type 2 diabetes (T2DM) and nonalcoholic fatty liver disease (NAFLD).^3^, ^4^ Consequently, therapeutic strategies to modulate the microbiome towards a more favorable profile have gained more interest in recent years.^5^ The short chain fatty acids (SCFA) that are produced from the microbial fermentation of indigestible carbohydrates (e.g., dietary fibers), often referred to as saccharolytic fermentation, can mediate diverse local as well as peripheral effects. These metabolites are put forward as the gateway through which the gut microbiome is able to affect host physiology and metabolism.^6^ The main three SCFA are butyrate, propionate, and acetate and are present in an estimated respective molar ratio of 20:20:60 in the colon and 4:5:91 in the systemic circulation.^7^, ^8^ All three SCFA have been recognized for their potential beneficial effects on metabolic health.^6^ Acetate, for instance, may have beneficial metabolic effects in context of obesity and glucose homeostasis.^9^ Although acetate is present at the highest concentration in intestine as well as systemic circulation, it is butyrate that has been under vigorous scientific scrutiny. Despite the extensive splanchnic extraction of butyrate, increased systemic butyrate concentrations in response to dietary fibers have been reported in healthy individuals^10^, ^11^ as well as individuals with metabolic syndrome (MetS).^12^ Its presumed anti‐inflammatory and weight‐reducing properties coined the idea that butyrate may act as a helpful tool for obesity control.^13^

However, the exact role of butyrate in the etiology of obesity remains controversial, since individuals with obesity appear to have higher fecal butyrate concentrations compared with their lean counterparts, even when a similar diet is consumed^14^, ^15^ and this difference is attenuated upon weight loss.^16^, ^17^ These observations have led some researchers to believe that butyrate may contribute to the obesogenic phenotype, for example, because microbial energy harvest from fibers is more efficient or because butyrate is used for de novo lipid synthesis.^18^, ^19^ Nevertheless, fecal concentrations may not accurately represent physiological concentrations because ˂10% of the total butyrate production is excreted in the feces. Mice studies suggest that the obese microbiota actually has a reduced capacity to ferment fibers^20^ and produce butyrate.^21^ Moreover, cross‐sectional data indicate an inverse association between fasting plasma butyrate and body mass index (BMI), pointing towards reduced circulating butyrate levels in individuals with obesity.^22^ The higher fecal butyrate levels observed in individuals with obesity may therefore merely reflect a difference in absorption or microbial utilization and not necessarily a higher production. Individuals with obesity or a disturbed glucose homeostasis actually seem to have a decreased abundance of butyrate‐producing taxa and a decreased expression of genes involved in butyrate production in the gut microbiome,^23^, ^24^, ^25^, ^26^ supporting a significant role for butyrate in energy and glucose homeostasis.

Whether the beneficial properties of butyrate can be translated to clinical practice and implemented to treat metabolic disturbances in humans still needs to be elucidated. Increasing colonic butyrate levels can be accomplished by various intervention strategies such as prebiotic and probiotic supplementation or transplantation of the intestinal microbiota.^13^, ^27^ Butyrate can also be administered as an end product itself either orally, intravenously, or rectally.^28^ These interventions may mediate differential effects considering it may reach different metabolically active organs. To illustrate, orally administered free butyrate is taken up almost entirely by enterocytes in the proximal intestine and may not reach the colon.^29^ Recently, an excellent review by Coppola et al.^30^ already highlighted the potential protective role of butyrate in obesity and obesity‐related disorders, predominantly by presenting animal data. Nevertheless, to exploit butyrate as a therapeutic intervention for obesity and disturbed glucose homeostasis in humans, it is crucial to characterize the conditions in which butyrate is (un)able to convey beneficial metabolic effects. Therefore, this review aims to assess the ability of butyrate to alleviate obesity‐related chronic low‐grade inflammation and impaired energy and substrate metabolism by integrating animal data with available human data to provide a comprehensive overview of the plethora of butyrate data that is out there. We summarize available literature on butyrate including its luminal production, absorption, and metabolism and discuss a mechanistic underpinning of its metabolic effects via interorgan cross talk. Thereafter, we discuss existing therapeutic strategies that aim to increase butyrate levels in the digestive system and/or the circulation and the current evidence regarding the putative effect of butyrate on body weight control and insulin sensitivity in humans. Lastly, this review intends to disentangle scientific inconsistencies and differences in the efficacy of human intervention trials to identify the hurdles that still need to be overcome in order to advance butyrate‐focused intervention aimed at improving metabolic health.

## 2 BUTYRATE: DIETARY SOURCES, LUMINAL PRODUCTION, AND KINETICS

### 2.1 Dietary sources of butyrate

Butyrate, a four carbon SCFA, is mainly formed from microbial saccharolytic fermentation in the colon and, to a minor extent, can also be produced from the fermentation of residual peptides or proteins (also referred to as proteolytic fermentation).^31^ Dietary fiber intake can lead to butyrate production in multiple ways: butyrogenic fibers increase butyrate production by acting as a substrate for bacterial fermentation, whereas bifidogenic fibers increase the abundance of bifidobacteria, which cannot produce butyrate themselves but increase butyrate production indirectly.^32^ Examples of dietary fibers that stimulate butyrate production include resistant starch and nonstarch polysaccharides such as arabinoxylans, β‐glucans, oligofructose, and inulin.^33^, ^34^, ^35^, ^36^ Resistant starch is naturally present in among others legumes, unripe bananas, and cooled‐down cooked potatoes but can also be added or fortified into bread and cereals.^34^, ^37^ Arabinoxylans are mainly found in wheat‐based products such a breakfast cereals and bread.^38^ Some of these breakfast cereals such as oats and barley may also contain β‐glucans, which is also naturally present in edible mushrooms and seaweed.^39^ Inulin can be found in a diverse set of plants and vegetables including Jerusalem artichoke, onion, and chicory root and is used as a fat replacer in many food products and, similar to oligofructose, can serve as replacement for sugar.^35^ Studies have shown that specifically resistant starch is potent in stimulating butyrate production and yields more butyrate compared with nonstarch polysaccharides.^40^, ^41^

Combining various fibers may provide a more optimal substrate or microbial environment for butyrate production than each fiber separately. To illustrate, a mixture of guar gum (propiogenic) and pectin (acetogenic) enhanced butyrate production in the caecum of mice after 6 weeks of supplementation.^42^ In general, the extent and rate of SCFA production from fibers depends on its fermentability, which can be influenced by numerous factors including: degree of polymerization, variations in esterification and saccharide linkage, the preparation method e.g., cooking and cooling,^37^ whether it is provided as a concentrate or in a whole‐grain matrix,^33^ and manufacturing methods e.g., entrapping the starch in microspheres.^43^, ^44^ Next to stimulating butyrate production, many of these fibers also influence other intestinal processes including alterations in intraluminal pH, gastric emptying, fecal bulking, and the production of bile acids along with systemic effects such as the feeling of fullness and direct effects on the immune system and glycaemic control.^45^ Although butyrogenic fibers may predominantly increase butyrate levels in the intestine, it usually also promote the production of other SCFA. Isotope tracing studies have revealed that inulin consumption, for example, significantly increased carbon enrichment of all three SCFA in the circulation in healthy individuals^46^ as well as individuals that were overweight or obese,^47^ although enrichment was highest for circulating butyrate. Hence, it is important to bear in mind that the beneficial metabolic effects of these fibers cannot be attributed to butyrate alone.^48^ Moreover, dietary intake and production of other SCFA can even potentiate the production and effect of butyrate itself. Functional metagenomic analysis showed an increase in butyrate production after resistant starch type 2 intervention in humans was predominantly dictated by the presence of *Ruminococcus bromii*, which produces the acetate necessary for butyrate production (as described in the following section).^49^ Furthermore, esterifying exogenous acetate to resistant starch, thereby delivering acetate to the colon, increased fecal and systemic butyrate concentrations and augmented weight loss and insulin sensitivity in obese mice compared with resistant starch alone.^50^

Butyric acid is also present in several food products that contain bovine milk fat, such as butter and cheese, in which the SCFA is esterified at the *α* (sn‐3) position.^51^, ^52^ This binding positioning in milk triacylglycerols strongly influences its catabolic rate since pancreatic lipase is able to cleave triacylglycerols at this position resulting in rapid free fatty acids (FFA) release in the small intestine.^53^, ^54^ Butyric acid can also be found in several triglyceride mixtures belonging to the short‐ and long‐chain acyl triglyceride molecule family. These food additives, also referred to as salatrims, are commonly used as a fat calorie replacer. In these mixtures, butyric acid is interesterified with a long chain fatty acid moiety such as stearic acid.^55^ In human clinical studies as well as rodent models for obesity and diabetes, butyrate is mainly supplied orally, in the form of sodium butyrate. Sodium butyrate is well‐known for its unpalatable flavor and odor and, since it does not require cleavage by lipase, is rapidly taken up in the upper gastrointestinal tract.^56^ At present time, novel strategies exist that improve the edibility and palatability of butyrate and/or increase the absorption and/or release of butyrate in the digestive tract. To illustrate, the use of a special coating made from hydroxy propyl methyl cellulose and Shellac on sodium butyrate tablets can delay its release in the intestinal tract by approximately 2 to 3 h, thereby delivering the product more distally.^57^ Furthermore, esterifying butyrate to a dietary fiber such as butyrylated starch prevents digestion in the upper part of the gastrointestinal tract and has shown to increase colonic butyrate concentrations in individuals with low^58^ and normal^59^ fecal butyrate concentrations. Tributyrin, in which butyrate is esterified to triglycerides, and other butyric acid derivatives such as 4‐phenylbutyric acid have an increased palatability and bioavailability compared with butyrate but may induce substantial side‐effects and therefore warrant caution if used in context of improving metabolic health.^60^

### 2.2 Butyrate biosynthesis

Two key bacterial strains are inferred with a capacity for butyrate production: *Faecalibacterium prausnitzii* (Clostridial cluster IV) and *Eubacterium rectale*/Roseburia *spp* (Clostridial cluster XIVa), both gram‐positive anaerobic bacteria belonging to the Firmicute family.^61^ Nevertheless, butyrate‐producing bacteria constitute a functional group rather than a specific phylogenetic family, as many other butyrate‐producing strains have been identified among various clostridial clusters.^18^, ^61^ The mildly acidic intestinal milieu in the proximal colon appears to promote butyrate‐producing bacteria, which thrive at a lower luminal pH, and thereby outcompete gram‐negative carbohydrate‐utilizing bacteria from the Bacteroides species.^62^, ^63^ Recently, an in vitro study using human fecal samples emphasized how colonic acidity can affect butyrate production. A pH ˃7.5 reduced the abundance of butyrate‐producing taxa, subsequently decreasing butyrate production, even when pectin was provided as a substrate.^64^

**FIGURE 1:**
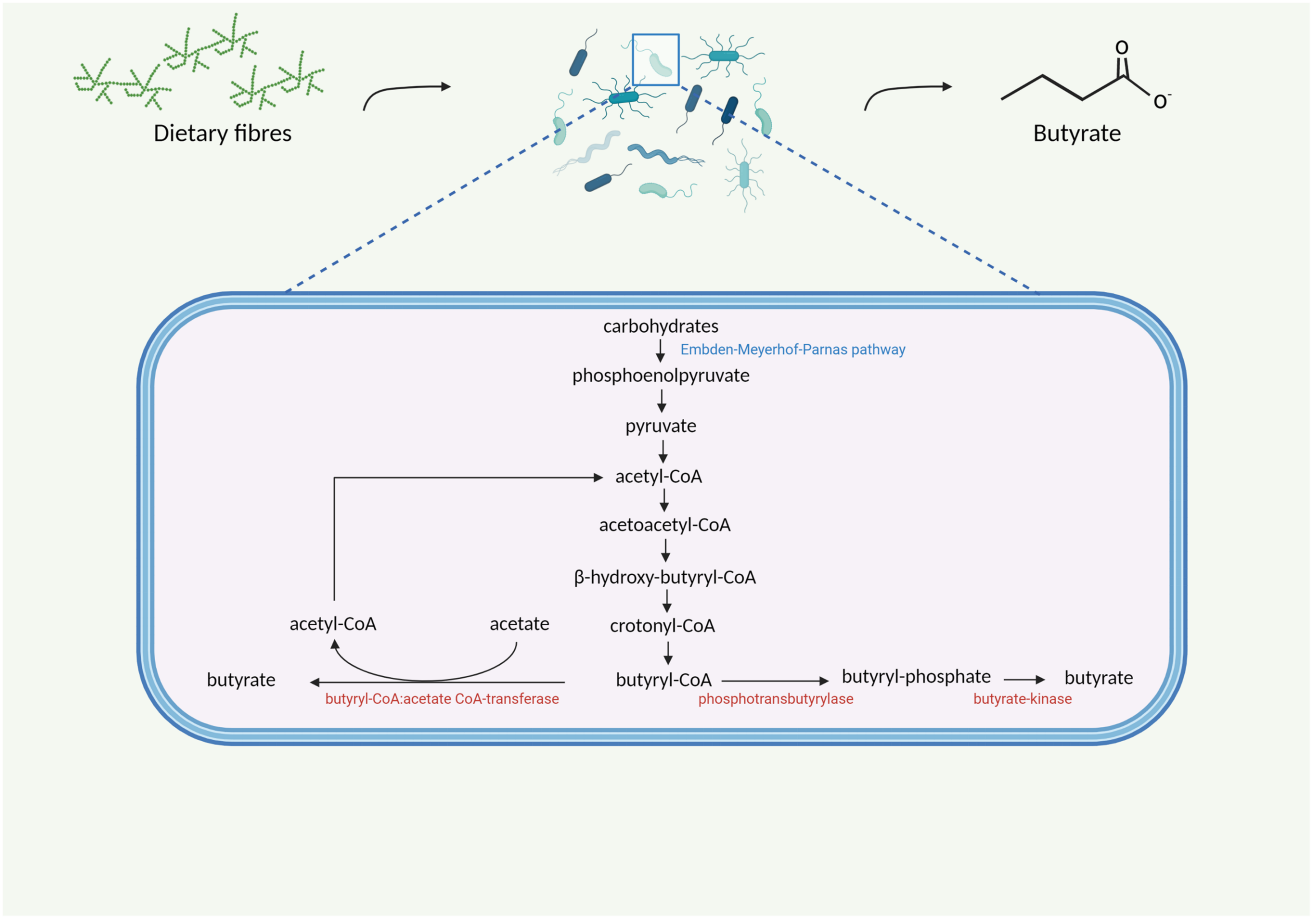
The microbial synthesis of butyrate in the colonic lumen. First, dietary fibers (carbohydrates) are broken down to monosaccharides and, subsequently, phosphoenolpyruvate through the Embden–Meyerhof–Parnas pathway. Thereafter, acetyl‐CoA is produced via pyruvate, which eventually gets converted to butyryl‐CoA. Butyryl‐CoA can be converted to butyrate through two pathways. The most common one uses acetate as a cosubstrate to generate butyrate and an acetyl‐CoA molecule and the other, less common, pathway, produces butyrate via butyrate‐phosphate. Both pathways are regulated by different enzymes (indicated in red in the figure). Created with BioRender.com

**FIGURE 2:**
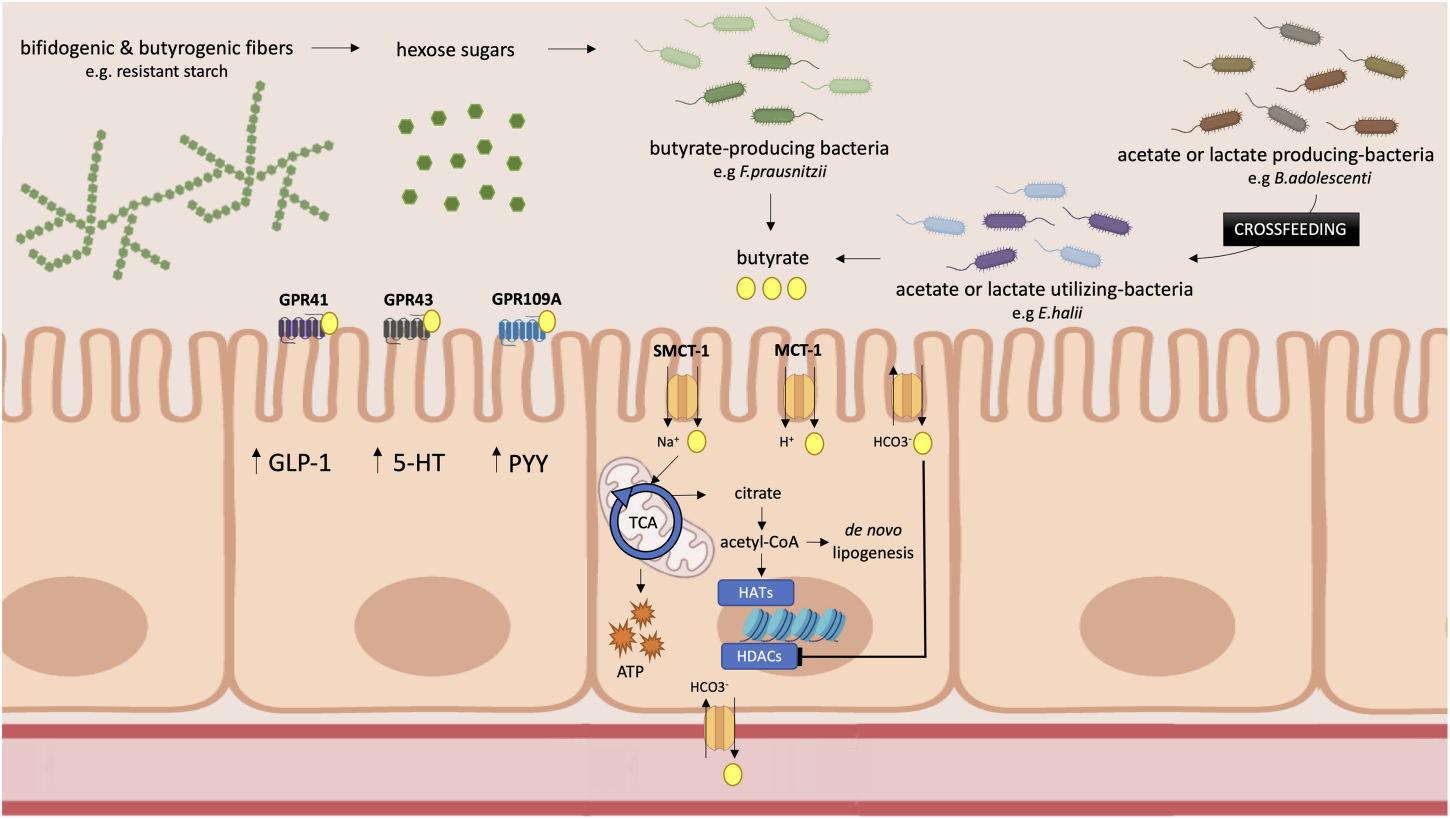
The production of butyrate from indigestible carbohydrates and its mechanism of action in the colon. Hexose sugars derived from complex indigestible carbohydrates are broken by specific bacterial strains and either produce butyrate directly (e.g., *Faecalibacterium* *prausnitzii,* *Eubacterium Rectale* , and *Roseburia intestinalis*) or via cross‐feeding pathways in which lactate or acetate is converted to butyrate. Butyrate is then absorbed into intestinal cells, predominantly by active transport systems including SMCT‐1 and MCT‐1. Thereafter, butyrate is either oxidized in the TCA cycle to generate ATP or cytosolic acetyl‐CoA is generated, which can be utilized for lipid synthesis or can activate HATs thereby influencing gene expression whereas intracellular butyrate can directly inhibit HDACs. 5‐HT, serotonin; acetyl‐CoA, acetyl coenzyme A; ATP, adenosine triphosphate; GLP‐1, glucagon‐like peptide 1; GPR109A, G protein‐coupled receptor 109A; GPR41, G protein‐coupled receptor 41; GPR43, G protein‐coupled receptor 43; HATs, histone acetylases; HDACs, histone deacetylases; MCT‐1, monocarboxylate transporter 1; PYY, peptide YY; SMCT‐1, sodium‐coupled monocarboxylate transporter 1; TCA, tricarboxylic acid cycle. Created with BioRender.com

Butyrate can be produced in the gut from hexose sugars by the condensation of by two acetyl coenzyme A (acetyl‐CoA) molecules. In postprandial conditions, the Embden–Meyerhof–Parnas pathway breaks down the hexose sugars derived from complex indigestible polysaccharides to produce phosphoenolpyruvate.^6^ Phosphoenolpyruvate acts as a precursor for acetyl‐CoA, which, by a succession of four rapid reactions, gets converted to butyryl‐CoA. The final step, transforming butyryl‐CoA into butyrate, can be performed by two different metabolic pathways, using different terminal enzymes: either phosphotransbutyrylase and butyrate‐kinase via butyryl‐phosphate or butyryl‐CoA:acetate CoA‐transferase (see Figure 1). The latter uses acetate as a cosubstrate and appears to be the most common pathway.^65^, ^66^ Metagenomic data indicate that these two acetyl‐CoA pathways together account for approximately 80% of total butyrate production, followed by the lysine pathway (11%). Glutarate and 4‐aminobutyrate, although only to a minor extent, can also serve as substrates for butyrate synthesis.^31^ Some strains including *Eubacterium hallii* and *Anaerostipes spp* have the ability to convert lactate or acetate into butyrate. Thus, some dietary fibers induce butyrogenic effects indirectly by increasing lactate or acetate production which in turn can be utilized by other bacteria to synthesize butyrate, a phenomenon referred to as cross‐feeding (see Figure 2).^32^, ^67^, ^68^, ^69^ Furthermore, a study comparing two in vitro gut models, one with both luminal and mucosal microbial niches and one without the mucosal niche, showed that the presence of a mucosal environment induced a shift from acetate towards butyrate production.^70^ This shift may be explained by certain butyrate‐producing strains that only adhere to the mucosal layer or because mucins, via cross‐feeding pathways, can act as a substrate for mucin‐converting microbes thereby generating acetate and lactate, which thereafter can be converted to butyrate.

### 2.3 Butyrate concentration in the human gut

The SCFA concentration along the gastrointestinal tract has two gradients: one from the proximal towards the distal colon and another from the base towards the top of the colonic crypt.^71^ Several studies have estimated that, on a daily basis, theoretically, 100–400 mmol SCFA can be produced from the consumption of 10 g of fiber.^72^, ^73^ Butyrate accounts for approximately 20% of the total SCFA production.^74^ The proximal colon, in particular the cecum, has the highest SCFA concentrations since the availability of substrates for saccharolytic fermentation is the highest here. As the availability of carbohydrate based‐substrates decreases towards the distal colon, SCFA concentrations decline and the amount of SCFA obtained from proteolytic fermentation increases. Proteolytic fermentation yields other by‐products besides SCFA including ammonia and branched SCFA such as isobutyrate and isovalerate.^75^ Hence, even though isobutyrate is an isoform of butyrate, it is formed from other substrates, mainly valine, by different microbial pathways and therefore may have distinct metabolic effects from butyrate. Isobutyrate is less readily absorbed and metabolized compared with butyrate but may act as an alternative energy source when butyrate levels are low or when butyrate oxidation is abberant.^76^ Little is known about the effect of branched SCFA on host health, but increased proteolytic fermentation has mostly been associated with detrimental health effects.^77^, ^78^, ^79^

Depending on the location in gastrointestinal tract and individual differences in dietary intake, gut transit time, and gut microbiome composition, colonic butyrate concentrations may vary, but it is estimated to range between 10 and 20 mmol per kg intestinal content.^80^, ^81^ Yet, lower estimations (1–10 mmol/L of intestinal content) have also been reported.^82^ Interestingly, recent work in animals and humans suggests that SCFA concentrations may fluctuate over the time span of a day.^83^, ^84^ Particularly later on the day, butyrate concentrations decreased as a result of a slight reduction in the abundance of several butyrate‐producing strains. These butyrate oscillations may be explained by eating behavior and meal timing, but other factors, independent of food intake, such as the level of circadian hormones may also play a role.^83^, ^84^ Interestingly, high fat diet (HFD)‐fed mice did not exhibit these diurnal butyrate fluctuation patterns, which indicates that microbial disturbances associated with the consumption of a westernized diet may disturb this circadian cycle of microbial butyrate production.^84^

### 2.4 Butyrate absorption, metabolism, distribution, and excretion

Butyrate absorption can occur in the small and large intestine via different routes (see Figure 2). In lipid‐soluble protonated form, butyrate is able to cross the apical membrane of the lumen through passive diffusion. However, since butyrate is a weak acid (*p*K ~4.8) and the colonic pH is between 5.5 and 6.7, >90% is present in ionized form and needs to be absorbed via an active transporter system.^85^, ^86^ Two main proteins involved in the transportation of anionic butyrate have been identified, both belonging to the monocarboxylate transporter family: the sodium‐coupled monocarboxylate transporter 1 (SMCT‐1)^87^, ^88^, ^89^ and monocarboxylate transporter 1 (MCT‐1).^90^, ^91^ SMCT‐1 is put forward as the primary butyrate transporter. As the name implies, its transport depends on the sodium gradient,^87^, ^88^, ^89^ whereas MCT‐1 transport is coupled to the proton gradient.^90^, ^91^ Butyrate can also be absorbed via a carrier‐mediated counter‐transport system that exchanges butyrate for bicarbonate, but, so far, the exact proteins responsible for this exchange remain unidentified.^92^, ^93^ Butyrate absorption may vary along the gastrointestinal tract as the expression of both transporters appears to increase from the jejunum towards the distal colon in the human intestine.^94^, ^95^ Sodium‐coupled butyrate transport probably plays a larger role in the distal colon as the SMCT‐1 K_m_ is considerably lower (~50 μM) than the MCT‐1 K_m_ (2.4–2.8 mM). The latter is therefore more active in the proximal colon where butyrate concentrations are high.^85^ Interestingly, evidence suggests that inflammation may decrease butyrate‐mediated uptake as well as the expression of both transporters.^95^, ^96^, ^97^ Thus, one may speculate that the inflammatory state associated with obesity may downregulate transporter‐mediated butyrate absorption. Limited literature is available on SCFA transport on the basolateral side of the membrane. Both SCFA‐bicarbonate exchangers and SCFA‐cation symport have been reported as plausible basolateral transport mechanisms. The kinetics of the SCFA‐bicarbonate antiporter on the basolateral and apical side differ, implying that the transport is managed by two different proteins.^93^, ^98^

After absorption, butyrate can be transported to the mitochondria for subsequent β‐oxidation. Here, butyrate is first converted back into butyryl‐CoA, which eventually yields two acetyl‐CoA molecules.^99^ In the initial step of the tricarboxylic acid cycle, acetyl‐CoA is converted to citrate which can by fully oxidized to generate adenosine triphosphate (ATP) or is shuttled out of the mitochondria and utilized for de novo lipogenesis.^71^ Because butyrate is the main oxidative substrate for colonocytes, accounting for more than 70% of their total energy demand,^100^, ^101^ concentrations in the portal vein are reduced by approximately 1000‐fold compared with colonic concentrations.^8^ Sudden death autopsies of six victims performed in the late 1980s revealed that butyrate concentrations in portal vein are approximately 29 μmol/L on average and decrease even further to 12 and 4 μmol/L in the hepatic and peripheral bloodstream, respectively.^8^ A more recent study determined SCFA flux in patients undergoing abdominal surgery and found a butyrate concentration of 30.1, 12, and 7.5 μmol/L in the portal vein, hepatic vein, and radial artery, respectively.^102^ Butyrate release appears highest in the distal intestine as butyrate concentrations were reported to be three times higher in the inferior mesenteric vein (approximately 62 μmol/L), which drains blood from the descending colon, sigmoid colon, and rectum, compared with the veins draining from proximal intestine (approximately 22 μmol/L).^103^ Another study showed that systemic butyrate concentrations rapidly declined after intravenous infusion and returned to initial values 1 h after administration, highlighting its short half‐life.^104^ More than 95% of butyrate is absorbed by the intestinal tract^105^ and for a large part is metabolized by enterocyte and colonocyte and thus excreted in expired breath in the form of CO_2._ ^106^ The remaining part (~5%) is excreted in the feces,^107^ and a negligible amount (<0.05%) can be traced back in urine.^104^, ^106^

### 2.5 Mechanism of action: HDAC inhibition and SCFA receptors

Many of the effects of butyrate are mediated through the activation of two intracellular pathways^18^ (see Figure 2). Firstly, butyrate is a histone deacetylase inhibitor (HDACi), specifically suppressing the activity of class I and II HDACs.^108^, ^109^ A HDACi inhibits the removal of acetyl groups from histones, making DNA more accessible for transcription and thereby increases the expression of downstream target genes.^109^ Several in vitro studies have shown that butyrate‐mediated HDAC inhibition may change T‐cell polarization and effector function including a shift from CD4+ naïve cells towards regulatory T‐cells^110^ and a shift in gene expression of Tc17 cells towards a more CD8+ cytotoxic T‐cell phenotype.^110^, ^111^ In this way, butyrate may regulate cytokine profiles, for example, by increasing the production of interleukin‐10 and interleukin‐17 and thereby decreases inflammation.^110^, ^111^, ^112^ Many of the tumor suppressive effects of butyrate, extensively reviewed elsewhere,^113^, ^114^, ^115^, ^116^ have also been attributed to HDAC inhibition.

Secondly, butyrate can bind to receptors belonging to the G protein‐coupled receptor (GPR) family (see Figure 2). All SCFA can bind to GPR41 and GPR43, but the receptor specificity varies per SCFA. Butyrate mainly activates GPR41 (ligand potency GPR41 = propionate = butyrate> acetate), whereas acetate and propionate prefer binding to GPR43 over butyrate (ligand potency GPR43 = propionate = acetate>butyrate)^117^, ^118^, ^119^, ^120^ albeit ligand specificity may be specie‐specific and appears different for mice and humans.^121^

The expression of GPR41 is widespread, most abundantly in adipose tissue^118^ and also in peripheral blood mononuclear cells,^117^ enteroendocrine cells, enterocytes,^122^ pancreas, spleen, bone marrow, and lymph nodes.^123^ Experiments conducted in knockout GPR41 mice suggest the receptor to be involved in peptide YY (PYY) release, intestinal transit rate, and energy harvest from the diet.^124^ GPR43 is predominantly expressed in immune tissues especially on polymorphonuclear cells such as neutrophils^118^, ^119^, ^125^ and also in skeletal muscle tissue, liver,^126^ white adipose tissue (WAT),^127^ and on serotonin‐containing mucosal mast cells and PYY‐releasing L‐enteroendocrine cells in the intestine.^128^ Hence, butyrate may stimulate intestinal PYY and serotonin release through GRP43 signaling. These L‐enteroendocrine cells simultaneously secrete proglucagon, which can act as a precursor for glucagon‐like peptide 1 (GLP‐1) production.^129^ GLP‐1 and PYY are gut‐derived hormones that influence insulin secretion and glucose homeostasis, therefore sometimes referred to as incretins, and also regulate food intake and satiety as circulating hormones and through innervation of the gut‐brain neural circuit.^130^ GPR43 knockout mice display weight gain, increased adiposity, and reduced systemic insulin sensitivity even on a normal chow diet, whereas adipose tissue‐specific GPR43 overexpression protects mice against the development of obesity even when a HFD is consumed.^131^ Both GPRs have been implicated with beneficial effects on intestinal barrier integrity, inflammation, and immunity thereby maintaining gut health.^132^, ^133^

Besides GPR41 and GPR43, butyrate is the only SCFA that can bind to GPR109A. This receptor is expressed in the small intestine, colon, adipose tissue, and several immune cells including macrophages.^134^, ^135^, ^136^, ^137^ In vitro work has shown that butyrate‐mediated GPR109A signaling promotes interleukin‐18 release from intestinal epithelial cells,^138^ inhibits nuclear factor ĸB signaling pathways in macrophages,^139^, ^140^ and reinforces colonic macrophages and dendritic cells to promote the differentiation of naïve CD4+ T cells into regulatory T‐cells and interleukin‐10 producing T‐cells,^110^, ^138^ which altogether reduce colonic inflammation. Interestingly, diabetic mice display increased GPR109A expression in the jejunum compared with nondiabetic controls. An explanation for this may be that GPR109A promotes glucose uptake, resulting in hyperglycemia.^141^ Recently, studies have unveiled that butyrate also binds to the olfactory receptor: Olfr558. Next to its function as olfactory sensory neurons in the nose cavity, this receptor is enriched in renal and cardiac vasculature and suggested to be involved in blood pressure regulation and muscle regeneration.^142^, ^143^

Thus, butyrate, as a HDACi, can directly influence gene expression and, through GPR activation, modulates appetite neurocircuitry and anti‐inflammatory immune responses. The production, absorption, metabolism, and mechanism of action of butyrate in the gut are summarized in Figure 2.

## 3 MECHANISTIC UNDERPINNING: BUTYRATE AND INTERORGAN CROSSTALK

### 3.1 Local intestinal and whole‐body effects

One of the primary functions of butyrate is to provide fuel to the cells lining the intestinal epithelium. Numerous studies have demonstrated that butyrate plays a crucial role in the energy homeostasis and mitochondrial functioning of colonocytes.^144^, ^145^, ^146^, ^147^ Colonocytes of germfree mice are energy‐deprived, but butyrate administration can restore this impaired mitochondrial respiration.^144^ Cytosolic acetyl‐CoA derived from butyrate via tricarboxylic acid cycle‐derived citrate can be utilized to form lipids or can transfer its acetyl groups to histone acetylases (HATs) (see Figure 2), which increase the expression of genes involved in cell proliferation and differentiation.^71^ Butyrate also appears to increase the expression of genes involved in fat and energy metabolism in human colonic mucosa.^148^ Additionally, butyrate plays an important role in maintaining gut health and gut functioning. It facilitates colonic transit and stimulates neuronal excitability of the colonic circular muscles,^149^, ^150^ presumably by promoting serotonin release, a well‐known stimulator of peristalsis.^151^ Butyrate can also promote intestinal gluconeogenesis in enterocytes through gene expression modulation.^152^ This butyrate‐induced gluconeogenic effect plays a significant role in its observed beneficial metabolic effects since butyrate administration was unable to enhance glucose tolerance or prevent weight gain in intestinal gluconeogenesis knockout mice.^152^

Besides its role in energy homeostasis, evidence suggests that oral butyrate supplementation modulates the composition and functionality of the gut microbiome^153^, ^154^, ^155^, ^156^, ^157^, ^158^, ^159^ and restores intestinal barrier integrity in diabetic as well as obese mice.^153^, ^158^, ^160^, ^161^, ^162^ Endotoxemia may play a crucial role in the chronic low‐grade inflammation observed in individuals with T2DM and/or obesity. Mice studies have shown an association between increased fat intake and endotoxemia, and this endotoxemia is associated with deteriorated glucometabolic parameters.^163^, ^164^ Human data seem to corroborate a relationship between intestinal leakage and metabolic health. People with T1DM and T2DM have significantly higher endotoxin levels than nondiabetic controls, which can be reduced by antidiabetic medication.^165^ Furthermore, a study showed that dietary fat intake acutely increased endotoxin levels in healthy individuals as well as individuals with obesity, yet a more pronounced elevation was observed in individuals with T2DM and obesity.^166^ A recent study reported a significant negative association between BMI and colonic permeability, and several “leaky” gut markers including zonulin were positively associated with metabolic health parameters in plasma.^167^ Butyrate may act as an intestinal barrier‐strengthening agent by regulating the expression, localization, and assembly of tight junction proteins^162^, ^168^, ^169^, ^170^, ^171^, ^172^ and promoting the production of antimicrobials^172^ and mucin glycoproteins^173^, ^174^, ^175^ and thereby could potentially counteract intestinal leakage. Nevertheless, these effects are mainly derived from animal and in vitro experiments and are not substantiated by human data yet.

Besides beneficial effects on the gastrointestinal barrier, butyrate stimulates the production of gut‐derived neuropeptides involved in energy homeostasis and food intake behavior such as glucose‐dependent insulinotropic polypeptide (GIP), GLP‐1, PYY, and serotonin in obese mice models.^176^, ^177^ In obese mice, acute intragastric butyrate administration but not intravenous butyrate administration significantly decreased 24 h food intake,^155^ implying that the anti‐obesity effect of butyrate is achieved by regulatory processes that occur before reaching the periphery. Human cross‐sectional data from a cohort covering individuals with a wide range of BMI and glucometabolic status demonstrated that fasting plasma butyrate concentration was significantly associated with circulating GLP‐1 but not PYY.^22^ Nevertheless, human experimental data remain limited. In patients with T2DM, 45 days of oral butyrate supplementation (600 mg/day) significantly increased serum GLP‐1 levels compared with placebo.^178^ Yet, acute rectal administration of SCFA mixtures, containing physiological amounts of butyrate, in men with overweight/obesity did not alter GLP‐1 but significantly increased fasting and postprandial plasma PYY concentrations.^179^ The effect of butyrate on the release of gut hormones warrants more investigation and may depend on intervention duration, mode of administration, and metabolic phenotype or pathological state of the sample population. To illustrate, a mice study comparing the effect of 12 weeks of supplementation with oral sodium butyrate, resistant starch, or a combination of the two reported that resistant starch (coincided by an increase cecal butyrate production) supplementation increased systemic PYY and GLP‐1 levels, whereas oral butyrate and the combination intervention did not alter or significantly decreased the levels of both incretins, respectively. These observations suggest that exogenous butyrate uptake in the upper GI‐tract may activate a negative feedback loop, thereby inhibiting incretin release from endogenous colonic butyrate.^180^

Despite low systemic butyrate concentrations, the effects of butyrate extend beyond the intestine. Animal work has shown that orally administered butyrate may increase energy expenditure,^181^, ^182^, ^183^ change systemic inflammatory marker profiles,^153^, ^160^, ^183^, ^184^, ^185^, ^186^ and alter energy substrate metabolism, promoting a shift from carbohydrate to fat utilization.^155^, ^182^ The weight‐reducing properties of butyrate are supported by abundant evidence from animal studies in which butyrate supplementation prevents diet‐induced weight gain.^153^, ^154^, ^155^, ^156^, ^158^, ^161^, ^177^, ^182^, ^183^, ^187^, ^188^, ^189^, ^190^, ^191^ In addition, animal studies using butyrate‐producing probiotic strains^192^, ^193^ or butyrogenic fibers^42^, ^50^ showed comparable beneficial results on weight control. Butyrate is known to act on the opioidergic system by epigenetically stimulating the expression of μ‐opioid receptor, which may be involved in reward‐related pathways that reduce food intake.^194^ Similarly, in obese rodent models, oral and intragastric butyrate administration improves insulin sensitivity and glucose tolerance.^155^, ^156^, ^181^, ^182^, ^183^, ^191^, ^195^ Besides the weight loss‐associated beneficial effects on glucose homeostasis, butyrate supplementation also attenuated oxidative stress and inflammation in nonobese diabetic mice and therefore may have additional antidiabetic properties besides weight loss.^185^ Nonetheless, the therapeutic effect of butyrate appears cohort‐dependent. To illustrate, the effects on inflammatory processes and intestinal homeostasis of monobutyrin (a glycerol ester of butyrate) treatment varied between two rat cohorts from identical strains kept under exactly the same experimental circumstances as a result of differential microbial composition and, subsequently, microbial metabolite production.^196^ Moreover, preliminary data indicate that obesity prone rats need a higher oral butyrate dose than obesity resistant rats (rats were categorized based on their weight gain after 8 weeks of HFD) to elicit the same response on body weight and glucometabolic parameters.^187^ Together, this emphasizes that microbial composition and metabolic phenotype can profoundly impact experimental outcomes.

Human evidence supporting the beneficial metabolic effects of butyrate remains limited. The proposed anti‐inflammatory potential of butyrate observed in animal studies is, for instance, not as evident in humans. A study evaluating the peripheral blood mononuclear cells of individuals with MetS after 4 weeks of daily oral 4 g sodium butyrate supplementation did not reveal overt effects on inflammatory cytokines production when stimulated by a diverse set of pathogenic stimuli.^197^ In contrast, butyrate intervention did significantly improve anti‐inflammatory response in the context of trained innate immunity, in which monocytes are capable of enhanced cytokine production upon secondary stimulation with an unrelated stimulus.^197^ Peripheral blood mononuclear cells of patients with T2DM that were supplemented 600 mg of sodium butyrate for 45 days displayed reduced markers for diabetes‐associated pyroptosis, a form of programmed cell death that promotes inflammation, compared with individuals that received placebo. Butyrate intervention upregulated the expression of several microRNAs that are known to inhibit inflammatory gene expression, potentially explaining this effect.^198^ Another study demonstrated that the incubation of monocytes, derived from patients with T2DM, with a supraphysiological butyrate concentration decreased monocyte migration and resulted in a more favorable tumor necrosis factor‐α/interleukin‐10 production ratio.^199^

**FIGURE 3:**
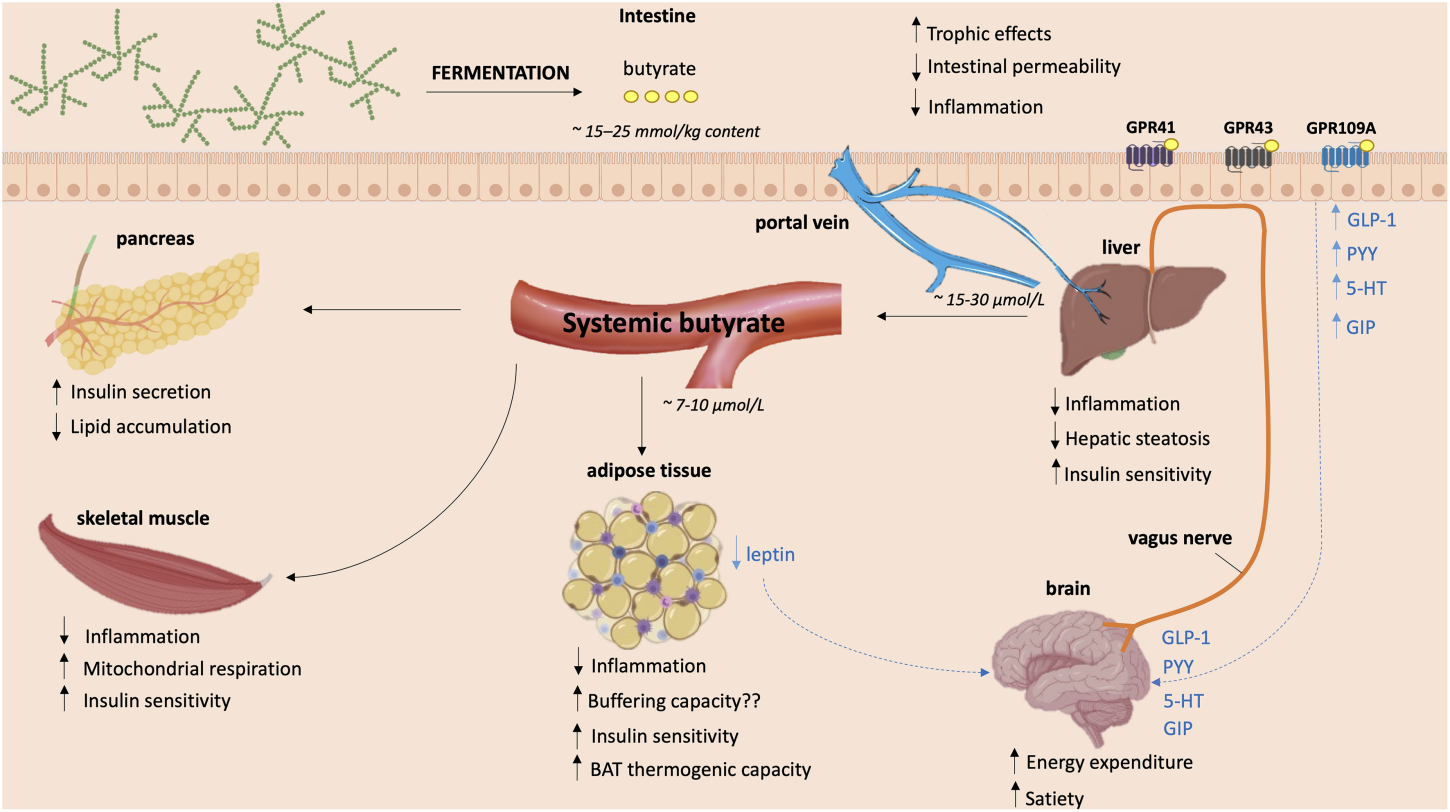
The putative metabolic effects of butyrate on different organs based on evidence from mainly animal and in vitro data. When butyrate is produced from the fermentation of dietary fibers in the intestine it mediates direct local effects on the intestinal barrier. Here, butyrate binds to G protein‐coupled receptors, thereby stimulating the synthesis of several neuropeptides that can signal to the brain. Butyrate that is not utilized by intestinal cells is transported to the liver via the portal vein. Thereafter, minor quantities of butyrate reach the circulation, consequently affecting other organs such as the adipose tissue, skeletal muscle tissue and pancreas. 5‐HT, serotonin; GIP, glucose‐dependent insulinotropic polypeptide; GLP‐1, glucagon‐like peptide 1; GPR109A, G protein‐coupled receptor 109A; GPR41, G protein‐coupled receptor 41; GPR43, G protein‐coupled receptor 43; PYY, peptide YY. Created with BioRender.com

In summary, butyrate is a pleiotropic metabolite that can induce a wide array of physiological functions and interorgan crosstalk may form the basis for its beneficial effects (see Figure 3). To compose a mechanistical framework, evidence regarding the effect of butyrate on insulin sensitivity and weight control on the liver, adipose tissue, skeletal muscle, pancreas, and brain will be discussed below.

### 3.2 Butyrate and liver metabolism

The first organ butyrate encounters after release from the intestine is the liver (except when butyrate is absorbed in the rectum region). Since hepatocytes extensively extract and metabolize butyrate, it may exert considerable effects here. Researchers have identified a liver‐specific butyrate transporter: organic anion transporter 7, which takes up butyrate in exchange for the sulfate‐conjugated steroid: oestrone sulfate. Consequently, hepatic butyrate transport may play a role in liver steroid hormone metabolism and detoxification processes.^200^ Additionally, an isotope tracing study revealed that butyrate infused in the caecum of mice can be traced back in the liver, where its carbon is incorporated into cholesterol and palmitate.^201^ Similarly, incubating isolated rat hepatocytes with butyrate‐induced cholesterolgenesis and lipogenesis.^202^ In human adolescents with obesity, plasma butyrate concentrations were associated with an increase in hepatic de novo lipogenesis after consumption of a high carbohydrate load.^203^ Together, these observations imply that butyrate may contribute to the accumulation of fat in the liver, also described as hepatic steatosis. Hepatic steatosis and hepatic inflammation are linked to the development of insulin resistance, a critical hallmark in the pathogenesis of T2DM and NAFLD. Intriguingly, in contrast to above indications that acute butyrate supplementation promotes hepatic steatosis, many studies report (chronic) that butyrate interventions mediate hepatoprotective effects in obese mice models^153^, ^155^, ^158^, ^181^, ^182^, ^183^, ^188^, ^195^, ^204^, ^205^ (see Figure 3). These butyrate‐fed mice exhibited significant reductions in the development of diet‐induced hepatic inflammation, intrahepatic fat accumulation, and liver injury.^153^, ^158^, ^182^, ^188^, ^204^, ^205^ However, a human study that evaluated intrahepatic triglyceride content by ^1^H‐liver magnetic resonance spectroscopy in individuals with MetS after 4 weeks of oral butyrate intervention did not observe any alterations in liver fat.^206^

A wide range of in vitro and in vivo studies have provided mechanisms by which butyrate may positively affect liver function and metabolism. First, a NAFLD mice model showed intragastric butyrate supplementation impeded HFD‐induced hepatic GLP‐1 receptor downregulation, which was independently associated with improved hepatic steatosis.^207^ Since hepatic GLP‐1 resistance may develop in patients with T2DM and NAFLD,^207^, ^208^ a GLP‐1 synthesizer like butyrate may work better than exogenous GLP‐1 or GLP‐1 agonists. Secondly, butyrate appears to avert diet‐induced hepatic proinflammatory cytokine and enzyme production in obese mice.^153^, ^205^ These anti‐inflammatory responses may partly be mediated by inhibiting an important pro‐inflammatory transcriptional regulator—nuclear factor‐κB.^205^ Thirdly, butyrate may increase the expression of nuclear factor erythroid 2‐related factor 2 and its downstream antioxidant enzymes including glutathione and thereby prevent diet‐induced hepatic oxidative stress.^183^, ^195^ Lastly, evidence suggests that butyrate may induce a switch from hepatic lipogenesis to β‐oxidation in obese mice, thereby improving hepatic insulin sensitivity.^182^, ^209^ This may be attributed to an effect on peroxisome proliferator activated receptor γ and fibroblast growth factor 21 (FGF21) expression, as butyrate was unable to convey beneficial hepatic effects peroxisome proliferator activated receptor γ^182^ and FGF21^209^ knockout mice. Butyrate activates FGF21 in vitro,^209^ and FGF21 overexpression in transgenic mice has shown to prevent diet‐induced obesity.^210^ FGF21 is a hepatokine (albeit also produced in minor amounts by other tissues such as skeletal muscle tissue) involved in the lipolysis and β‐oxidation of long‐chain fatty acids^209^ and may upregulate GLUT1 expression and glucose uptake in extrahepatic tissues such as the adipose tissue.^210^ Nevertheless, increased levels of serum FGF21 are reported in individuals with obesity and/or T2DM,^211^, ^212^ suggesting FGF21 resistance may have developed over time. Indeed, clinical trials using FGF21 analogs in individuals with obesity did not show any weight‐reducing effects although lipid profiles, glucose homeostasis, and whole‐body insulin sensitivity were improved.^213^, ^214^

Ex vivo experiments suggest that butyrate promotes hepatic gluconeogenesis^215^, ^216^ and has adverse effects on hepatic mitochondrial energy homeostasis.^217^, ^218^ However, these observations do not translate to the in vivo situation. In diabetic mice, butyrate supplementation reduced gluconeogenesis, glycated hemoglobin (HbA_1c_), and insulin resistance^219^ and these restorative effects on hepatic glycolipid metabolism and liver histology are supported by numerous other studies using diabetic mice.^157^, ^160^, ^161^, ^220^, ^221^ Animal studies showed that administration of sodium butyrate improved hepatic mitochondrial dynamics and efficiency,^183^ increased phosphorylation of the AMP‐activated protein kinase/acetyl‐CoA carboxylase pathway,^183^, ^207^ and increased expression of glucose transporter 2^183^ and the insulin receptor,^207^ which may explain improved hepatic insulin sensitivity.

Altogether, an intriguing amount of animal data suggests that butyrate ameliorates diet‐induced hepatic insulin resistance, fat deposition, and inflammation, whereas human data are lacking. Future studies should use ultrasound‐based technology and magnetic resonance imaging techniques to assess liver histology and the amount and distribution of liver fat after butyrate‐focused interventions in humans.^222^

### 3.3 Butyrate and adipose tissue metabolism

One key function of the adipose tissue is to store dietary fatty acids in the postprandial state, to be released in times of increased energy requirement. In individuals with obesity and insulin resistance, adipose tissue functioning appears impaired. This dysfunction is characterized by immune infiltration, a reduced storage capacity, and lipid spillover, contributing to systemic low‐grade inflammation and ectopic fat accumulation, respectively, which eventually disturbs insulin signaling.^223^ Adipocytes may interact with macrophages and other immune cells, and this interaction may contribute to the observed chronic low‐grade inflammation.^224^, ^225^ Butyrate supplementation has shown to attenuate diet‐induced adiposity in obese^153^, ^155^, ^181^, ^226^, ^227^, ^228^ as well as (pre)diabetic rodent models^161^, ^219^ and may reduce adipocyte hypertrophy associated with a HFD.^155^, ^182^, ^189^, ^228^, ^229^ Moreover, protein analysis of the adipose tissue of butyrate‐fed obese mice demonstrated increased expression of the insulin receptor^189^ as well as downstream targets such as glucose transporter 4^156^, ^189^ compared with HFD‐controls, suggesting improved adipose‐tissue insulin sensitivity (see Figure 3). Butyrate is proposed to influence adipose tissue function in several ways, by affecting intracellular adipogenesis, lipolysis, and adipose tissue inflammation.

In vitro and in vivo work propose that butyrate triggers adipocyte hyperplasia by stimulating adipogenesis.^189^, ^230^ This effect is supported by increased expression of adipose tissue‐specific proliferating cell nuclear antigen, an essential protein for DNA replication, in butyrate‐treated versus control‐fed obese mice.^189^ In contrast, in lean mice and piglets, adipogenesis appears reduced after long‐term butyrate treatment,^231^, ^232^ suggesting that butyrate‐induced alterations in adipogenesis may differ in lean and obese animal models. The effect of butyrate on lipolysis still remains under debate as some studies suggest it stimulates lipolysis, whereas others report antilipolytic effects. Several in vitro studies suggest that supraphysiological^230^, ^233^, ^234^ as well as physiological butyrate concentrations increase basal and β‐adrenergically mediated glycerol release, which is a measure for adipose tissue lipolysis.^234^ Butyrate may mediate lipolytic effects through gene modification.^233^ Specifically increased acetylation and activation of the β3‐adrenergic receptor, a key regulator in lipolysis, have been reported after butyrate intervention in the WAT of obese mice.^228^ Evidence suggests that β‐adrenergic‐mediated lipolysis is blunted in context of obesity which may, among other things, be explained by a reduced level and sensitivity of the β3‐adrenergic receptor.^235^, ^236^ Hence, if butyrate‐mediated activation of the β3‐adrenergic receptor also occurs in humans, this may potentially (partially) restore sensitivity. Yet, in contrast to the increased lipolysis after incubation with butyrate alone, a SCFA mixture high in butyrate concentration (35%) did not affect basal nor β‐adrenergically mediated glycerol release in a human adipocyte model.^234^ Moreover, opposed to lipolytic effects in monoculture, butyrate appears to diminish lipolysis concurrent with reduced inflammatory responses in a differentiated adipocyte‐macrophage co‐culture.^140^, ^237^ Thus, it is crucial to evaluate adipocytes in context of macrophages. These adipocyte interactions highlight that in vivo studies need to be conducted in order to investigate the effects of butyrate on adipose tissue in context of other tissues (as they may affect one another). In obese rodent models, chronic butyrate treatment attenuated diet‐induced elevations in systemic lipid profiles including triglycerides and cholesterol.^155^, ^181^, ^187^, ^188^, ^190^, ^191^, ^238^ These improved lipid markers hint towards enhanced adipose tissue storage capacity but may also be the result of improved liver functioning (or both). Human cross‐sectional data showed that fasting plasma butyrate levels were negatively associated with plasma FFA levels. Yet, no significant associations were observed with plasma triacylglycerols and glycerol.^22^ Rectal administration of a SCFA mixture containing high butyrate concentrations significantly increased lipid oxidation and reduced fasting plasma free glycerol compared with placebo in men that were obese or overweight.^179^ However, this increase in lipid oxidation was significantly correlated to plasma acetate but not to butyrate concentrations. A 4week intervention study in individuals with MetS from both sexes showed 4 g/day of oral butyrate supplementation significantly reduced total cholesterol and triglycerides levels compared with baseline.^206^ In contrast, another comparable study with men with MetS observed a significant increase in plasma total cholesterol and low‐density lipoprotein cholesterol and no alterations in FFA and triglycerides compared with initial levels.^159^ In patients with T2DM receiving 600 mg/day for 6 weeks, a similar increase in plasma total and low‐density cholesterol was observed albeit only compared with baseline levels and not compared with placebo.^239^

Besides adipogenesis and lipolysis, butyrate also alters the expression of proteins involved in adipose tissue inflammation, also referred to as adipokines.^240^ Butyrate administration has shown to attenuate the production of several diet‐induced pro‐inflammatory markers including tumor necrosis factor‐α in the adipose tissue of obese mice^153^, ^158^, ^162^ and diabetic mice.^241^ Additionally, evidence from obese mice models suggests that chronic butyrate supplementation decreases systemic and adipose tissue‐specific leptin^177^, ^183^, ^189^, ^227^, ^228^, ^229^ and increases adiponectin^183^, ^189^ concentrations, two other well‐known adipokines, towards a similar range as those of lean mice. Leptin is associated with inflammatory processes, increasing in proportion to body fat, whereas adiponectin has an inverse relationship with adipocyte size and may contribute to anti‐inflammatory processes, adipose tissue vascularization, and insulin sensitivity.^242^, ^243^ Recent work in an obesity mice model also showed that sodium butyrate supplementation may reinforce a more anti‐inflammatory immune cell profile in the adipose tissue, shifting towards increased levels of M2 macrophages and regulatory T‐cells relative to the population of M1 macrophages and naïve CD4+ T‐cells.^162^

Next to effects on the WAT, butyrate treatment may stimulate mitochondrial activity, lipid oxidation, and thermogenic capacity, evidenced by elevated uncoupling protein‐1 protein levels, in the brown adipose tissue (BAT) of obese^155^, ^181^ and microbiota depleted mice.^244^ Additionally, BAT and subcutaneous WAT may metabolize butyrate because the adipocytes of butyrate‐treated mice exhibit increased mRNA acyl‐CoA medium‐chain synthetase 3 expression, the enzyme for the initial step of butyrate oxidation, and carnitine palmitoyltransferase 1α expression, suggesting elevated fatty acid oxidation.^245^ These effects may underpin butyrate's presumed beneficial effect on energy expenditure in animal models. Nevertheless, 4 weeks of 4 g/day butyrate intervention did not alter metabolic BAT activity or resting energy expenditure in lean men nor men with MetS.^159^

Overall, animal studies suggest that butyrate may restore adipose tissue inflammation and activate BAT. Yet, its effect on lipogenesis and lipolysis remains inconsistent, and human data, so far, do not solidify the observations of animal studies.

### 3.4 Butyrate and skeletal muscle metabolism

Skeletal muscle may account for approximately 80% of the insulin‐stimulated glucose clearance under hyperinsulinemic‐euglycemic clamp conditions.^246^, ^247^ In postprandial conditions, this is considerably lower, accounting for 23% of total glucose disposal^248^, ^249^ but still plays an important part in regulating energy flux. The obese insulin‐resistant phenotype is characterized by impaired mitochondrial functioning and metabolic inflexibility, in which the skeletal muscles can no longer match lipid oxidation to the increased lipid supply.^223^, ^235^ Moreover, both T2DM and obesity have been associated with relative loss of muscle mass and strength.^250^ Few studies have investigated the specific effect of butyrate on muscle metabolism, but sodium butyrate treatment has shown to reduce lipid accumulation^191^, ^227^ and improve mitochondrial functioning in the skeletal muscle of obese rodents^181^, ^187^, ^227^ (see Figure 3). These effects might be mediated by increased expression of antioxidant enzymes and peroxisome proliferator‐activated receptor γ isoform α and mitochondrial transcription factor A, two transcriptional regulators involved in mitochondrial biogenesis.^187^, ^232^ Additionally, chronic butyrate interventions have shown to increase the percentage of slow‐twitch type I muscle fibers in obese mice^181^, ^226^ and lean piglets.^251^ These fibers are oxidative and contain more mitochondria than fast‐switch type II muscle fibers. Short‐term butyrate supplementation may enhance mitochondrial lipid oxidation in the gastrocnemius muscle of obese mice, indicated by increased expression of genes and proteins involved in lipid oxidation and oxidative phosphorylation compared with control.^181^, ^227^ A mice model investigating the effect of chronic butyrate administration in aging mice supports above reported effects as butyrate‐reduced intramuscular fat accumulation and increased markers of mitochondrial biogenesis, antioxidant activity, and oxidative metabolism in the skeletal muscle.^252^ A human cross‐sectional study using mendelian randomization analysis has identified a causal relationship between the production of microbial butyrate and appendicular lean mass in Chinese menopausal women, suggesting that butyrate may play a role in maintaining muscle mass in humans as well.^253^

Butyrate may also increase muscle‐specific insulin sensitivity,^181^, ^187^ evidenced by enhanced phosphorylation of the insulin receptor substrate 1^181^ and increased mRNA expression of insulin receptor substrate 1 and glucose transporter 4^187^ in the gastrocnemius muscle of butyrate‐treated obese rodents compared with controls. Nevertheless, the insulin‐sensitizing effect of butyrate is probably also mediated indirectly, via the production of gut‐derived incretins. GLP‐1 is known to alter muscle microvasculature increasing both blood volume and blood flow in insulin sensitive healthy humans^254^ and rats,^255^ and these responses remain preserved in insulin‐resistant rats.^256^ In this way, butyrate may enhance insulin action and glucose oxidation in the muscle because insulin delivery is increased as a result of enlarged endothelial myocyte surface. Furthermore, incubating primary myocytes derived from individuals with obesity with GLP‐1 increased glucose uptake and restored the activity of enzymes involved in muscle metabolism.^257^ Similar effects have been reported for PYY.^258^

Altogether, butyrate may counteract obesity‐associated mitochondrial dysfunction and muscle atrophy and can indirectly increase insulin‐mediated glucose disposal in the muscle tissue. Future butyrate‐focused intervention studies in humans should evaluate transcriptomics from muscle biopsies and changes in muscle mass, for example, by a dual X‐ray absorptiometry scan.^259^

### 3.5 Butyrate and pancreatic insulin functioning

The pancreas is a crucial organ for energy and substrate metabolism, responsible for among others the secretion of insulin, a key hormone in the regulation of postprandial substrate metabolism. Butyrate might be able to prevent pancreatic dysfunction associated with the insulin‐resistant obese phenotype. Animal data indicate that butyrate may increase insulin secretion^161^, ^177^, ^187^ and reduce pancreatic fat deposition and β‐cell damage, thereby preserving islet functioning^161^, ^187^, ^190^, ^219^, ^238^ (see Figure 3). Several studies have shown that chronic butyrate treatment decreased fasting insulin levels in T2DM rats^260^ and obese rodents^156^, ^177^, ^183^, ^191^, ^238^ compared with their respective controls. One of these studies showed that acute butyrate administration rapidly increased insulin release compared with a saline control, whereas the same dose of other fatty acids including acetic acid did not significantly alter insulin secretion.^177^ In vitro studies performed a couple of decades ago suggest that butyrate induces an acute stimulatory effect on insulin release.^261^, ^262^ Nevertheless, these studies used supraphysiological concentrations (2–10 mM), and recent work with rat islets only demonstrated a significant effect on pancreatic β‐cell functioning after 24‐h incubation with 5 mM of sodium butyrate, whereas an acute insulinotropic effect was not observed.^263^ Since pancreatic β‐cells express GPR41 and GGPR43,^264^ butyrate may directly regulate insulin secretion through G protein mediated signaling, yet whether this occurs remains controversial.^265^ The observed insulin release pattern after acute butyrate administration in HFD mice overlapped with GLP‐1, PYY, and GIP release, while other SCFA were unable to induce gut‐derived hormones (with exception of propionate‐induced GIP stimulation), pointing towards indirect regulation of insulin production. GLP‐1 can influence pancreatic β‐cells by accelerating the glucose‐dependent closure of ATP‐regulated potassium channels, which provokes postprandial insulin secretion^266^ and simultaneously inhibits glucagon release.^267^ Moreover, butyrate may also stimulate the antioxidant defense system in the pancreas^187^ and inhibit pancreatic β‐cell apoptosis through gene expression modulation^187^, ^190^, ^260^ thereby indirectly contributing to enhanced pancreatic functioning.

Altogether, animal data suggest that butyrate may potentially improve pancreatic insulin response but the acute effect of butyrate on glycaemic control and insulin release (in dietary context) remains to be investigated in humans. Future studies could study the effect of different doses of butyrate on postprandial substrate metabolism by using a cross‐over design.

### 3.6 Butyrate and the brain

The brain plays an important regulatory role in energy homeostasis as a master regulator of food intake behavior and serving as a thermostat for energy expenditure. Evidence suggests that obesity is characterized by vagal afferent signaling dysregulation, indicated by a diminished ability to switch off orexigenic responses in the fed state as well as a reduced sensitivity to endocrine satiety proteins.^268^ Moreover, besides the commonly known obesity‐associated metabolic complications, obesity is associated with neuropathic pain, alterations in brain structure, impaired cognitive functioning, and increased risk of developing neurogenerative diseases including Alzheimer's.^269^, ^270^ Mice studies have demonstrated that butyrate may have the ability to counteract these obesity‐associated neurological changes,^188^, ^271^, ^272^ and the pain‐reducing properties of butyrate have been corroborated by a cross‐over randomized controlled trial (RCT) using rectal butyrate enemas in healthy adults.^273^

As systemic butyrate levels are relatively low, it is unlikely that note‐worthy amounts of butyrate reach the brain. Positron emission tomography in nonhuman primates confirmed that butyrate can cross the blood barrier, but uptake is extremely low (˂0.006%).^274^ In contrast, substantial increases in butyrate concentration in the brain were reported after administration of butyrate‐producing bacterial strains in mice,^275^, ^276^ and an isotope tracing study in mice suggests that butyrate contributes to tricarboxylic acid metabolites in the brain.^244^ Despite these observations, the effect butyrate may have on the brain is probably predominantly indirect. Butyrate may act as a sensor to provide intestinal information to the brain by signaling brain regions involved in food intake including the nucleus tractus solitaries. A mice study demonstrated supraphysiological intraperitoneal butyrate injection (1–6 mmol/kg) dose‐dependently and time‐dependently decreased food intake and induced the strongest anorexigenic effect out of the three major SCFA. This anorexigenic effect was completely abolished by capsaicin pretreatment, an inhibitor of afferent vagal nerve innervation. Selective inhibition of the hepatic branch of the vagus nerve resulted in a similar inhibitory effect, so a hepatic–portal–vagal route may be at play. The authors postulated butyrate may regulate satiety via GPR41 or GPR109A signaling on nodose ganglion neurons in the brain,^277^ but this remains to be investigated.

As stated previously, butyrate stimulates the production of gut‐derived neuropeptides including serotonin, GIP, GLP‐1, and PYY as well as adipose tissue‐derived leptin. Similarly, monobutyrin supplementation preserved the sensitivity to cholecystokinin, another well‐known vagal anorexigenic stimulator, and strengthened the response to cholecystokinin‐induced energy intake reduction in HFD‐fed mice.^196^ These endocrine proteins and neurotransmitters can signal various hypothalamic nuclei in the brain resulting in an increased feeling of satiety and a reduced drive to eat^130^, ^278^, ^279^ (see Figure 3). Intriguingly, a mice study showed intragastric butyrate administration but not intravenous administration led to a significant decrease in 24‐h food intake compared with control, and this was abolished by subdiaphragmatic vagotomy. Intragastric butyrate supplementation reduced FOS‐positive neurons, a marker for neuronal activity, in the nucleus tractus solitaries and dorsal vagal complex in the brainstem and reduced c‐FOS expression of neuropeptide Y positive orexigenic neurons in the hypothalamus.^155^ Taken together, this study suggests that the gut–brain axis is necessary for butyrate to elicit a significant effect on food intake behavior.

Both WAT and BAT depots interact with the brain through distinct sympathetic neuronal axon projections (and the WAT also via leptin production). Whereas the WAT is predominantly involved with energy storage, the BAT may modulate energy expenditure.^280^ Butyrate‐fed mice exhibit elevated tyrosine hydroxylase expression in the BAT, a marker for peripheral sympathetic nerve activity, compared with control obese mice. The butyrate‐induced thermogenic effect and increased lipid oxidation observed in the BAT were diminished after vagotomy, which also points towards (partial) regulation by the vagus nerve.^155^ However, the quantification of metabolically active BAT and its contribution to energy expenditure in humans remains uncertain and sympathetically mediated thermogenesis is probably predominantly generated by skeletal muscle tissue.^281^, ^282^

How butyrate may affect the activity of reward‐related pathways in humans remains to be investigated. Clinical studies have demonstrated that 4 weeks of 4 g/day sodium butyrate did not alter total energy intake compared with the start of the intervention in lean individuals,^159^ patients with T1DM,^283^ nor individuals with MetS.^159^, ^206^ One of these studies also evaluated if butyrate had any satietogenic effects, by using the Visual Analog Scale for appetite and hunger, but no changes were observed post intervention.^206^ Despite these findings, the same study revealed that butyrate supplementation modulated neural pathways in the brain. Butyrate intervention had a tendency to reduce cerebral dopamine transporters binding in the striatum of individuals with MetS.^206^ This transporter has been linked to reward processing and glucose homeostasis and appears downregulated in people with a higher BMI. Hence, a reduced dopamine transporter binding may appear counterintuitive since butyrate is considered an anorexigenic stimulator. Heart rate variability, a marker of autonomic nervous system activity, was also significantly increased post butyrate intervention.^206^ Both observations advocate butyrate can affect the human brain dopaminergic system and vagal nerve innervation, respectively. However, additional research is required to elucidate how these pathways are affected and if they translate to actual dietary changes in humans.

Overall, animal data suggest that butyrate may provide a therapeutic strategy to innervate the central nervous system and combat obesity‐associated impaired sympathetic signaling. Yet, reductions in food intake or satiety in response to chronic butyrate intervention in humans have not been reported so far. A summary of the effects of butyrate derived from animal studies on organ level and the mediated crosstalk between organs is displayed in Figure 3.

## 4 HUMAN BUTYRATE‐FOCUSED THERAPEUTIC INTERVENTIONS TO TREAT OBESITY AND RELATED METABOLIC DISORDERS

From a mechanistic perspective, abundant evidence from animal and cell models suggests that butyrate has putative beneficial effects on metabolic health and the function of peripheral tissues. Nevertheless, the question remains whether this can be translated to a useful intervention strategy for humans. For this purpose, this section will evaluate the efficacy of butyrate‐focused interventions on metabolic health in humans. Clinical studies modulating the gut microbiome were only included if they increased a butyrate biomarker, for example, butyrate‐producing microbial strains, fecal, and/or plasma butyrate concentrations.

### 4.1 Gut microbial modulation, body weight control, and glucose homeostasis

A pilot study with men with MetS (*n =* 18) implicated that butyrate may play a significant role in the changes in insulin sensitivity observed after fecal microbial transplantation (FMT) (see Table 1). A single dose of FMT from a lean donor (allogenic transplantation) significantly increased the abundance of butyrate‐producing strains *Roseburia intestinalis* and *Eubacterium hallii*. Concurrently, peripheral insulin sensitivity, measured by the golden standard hyperinsulinemic‐euglycemic clamp, increased slightly but significantly and hepatic insulin sensitivity had a tendency to improve from baseline albeit not compared with placebo (autologous transplantation). Other metabolic parameters such as BMI, fasting glucose levels, and HbA_1c_ remained unaltered compared with baseline. Despite increased levels of butyrate‐producing strains, fecal total SCFA and butyrate concentrations decreased after FMT yet were maintained after autologous transplantation.^284^ In line with these results, a follow‐up study performed with a larger sample size of men with MetS (*n =* 44); demonstrated FMT indeed significantly increased peripheral insulin sensitivity.^291^ However, these effects were transient, returning both microbial composition and insulin sensitivity to initial state after 18 weeks, and the authors attributed the metabolic alterations to other metabolites than butyrate including an increase in fecal acetate. Moreover, a large variation in FMT‐induced glucometabolic response was observed depending on initial microbiota composition.^291^

**TABLE 1:** The effect of intervention strategies aimed at increasing microbial butyrate production on weight and glucose metabolic status

Participants	Intervention	Design, *duration*, and frequency	Metabolic effects	Study
Males with metabolic syndrome (*n =* 18)	Allogenic FMT (from lean male donors; *n =* 9) or autologous FMT (reinfusion of own feces; *n =* 9)	RCT *Outcomes measured after 6 weeks* Single dose	**↑** Butyrate‐producing strains in gut microbiome **↓** All fecal SCFA concentrations including butyrate **↑** Peripheral insulin sensitivity and tendency to **↑** hepatic insulin sensitivity compared with baseline but not placebo	Vrieze et al. (2012)^284^
Males with metabolic syndrome (*n =* 24)	*A. soehngenii* administration with low (10^6^ cells/ml, *n =* 8), medium (10^8^ cells/ml, *n =* 8), high dose (10^10^ cells/ml, *n =* 8)	Randomized trial *4 weeks* 1x/day	**↑** Fecal butyrate‐producing *A.soehngenii* with greatest effect in highest dose No differences in fecal SCFA levels No significant difference in peripheral insulin sensitivity between groups	Gilijamse et al. (2020)^285^
Individuals with T2DM (*n =* 58)	WBF‐010 (consisting of inulin, *C. beijerinckii, C. butyricum, B. infantis*; *n =* 21) or WBF‐011 (consisting of inulin, *A. muciniphila, C. beijerinckii, C. butyricum, B. infantis and A. hallii*; *n =* 21) or only colloidal silicon dioxide (placebo; *n =* 16)	RCT *12 weeks* Dose divided in 2x/day	**↑** Butyrate‐producing *A.halli* more frequently detected in stool samples after 4 and 12 weeks of WBF‐011 No significant changes in fecal SCFA but **↑**fasting plasma butyrate **↑**plasma butyrate associated with **↓**HbA_1c_ in participants who were not taking a sulfonylurea agent WBF‐011 significantly **↓** AUC and IAUC of total glucose concentration during a meal‐tolerance test WBF‐011 had a tendency to **↑** postprandial insulin secretion and**↓**HbA_1c_ No changes in HOMA‐IR, fasting glucose, and body weight	Perraudeau et al. (2020)^286^, ^287^
Individuals with T1DM (*n =* 18)	40 g of type 2 resistant starch consisting of a high‐amylose (70%) maize starch with acetate and butyrate attached to it	Single arm pilot study *6 weeks + follow‐up at week 12* 1x/day	**↑** Fecal and circulating butyrate and acetate after 6 weeks No alterations in HbA_1c_, insulin dosage, and mean daily average blood glucose Circulating butyrate at week 6 was negatively associated to HbA1_c_, % of time below target range (< 3.9 mmol/L), and basal insulin dose	Bell et al. (2022)^288^
Females with obesity (*n =* 30)	16 g of inulin‐type fructans prebiotics (a 50/50 mix of inulin/oligofructose; *n =* 15) or maltodextrin placebo (*n =* 15)	RCT *3 months* Dose divided in 2x/day	**↑** Butyrate‐producing *F*. *prausnitzii* in gut microbiome **↓** AUC of total glucose concentration after oral glucose tolerance test No alterations HbA_1c_, HOMA, and fasting insulin No changes in BMI and hip‐waist‐ratio but slight tendency to **↓** fat mass	Dewulf et al. (2012)^289^
Healthy individuals (*n =* 35)	16 g of FOS (*n =* 34) or GOS (*n =* 35) prebiotics	Cross‐over randomized trial *14 days* Dose divided in 2x/day	**↓** Butyrate‐producing strains in gut microbiome FOS significantly **↓** (46.1%) fecal butyrate and worsened postprandial glucose response GOS had a tendency to **↓** (31.2%) fecal butyrate and significantly decreased fasting glucose levels	Liu et al. (2017)^290^

Abbreviations: AUC, area under the curve; BMI, body mass index; FMT, fecal microbial transplantation; FOS, fructo‐oligosaccharides; GOS, galacto‐oligosaccharides; HbA_1c_, glycated hemoglobin; HOMA‐IR, homeostatic model assessment for insulin resistance; IAUC, incremental area under the curve; RCT, randomized controlled trial; SCFA, short chain fatty acids.

Instead of transferring the entire microbiota, specific butyrate‐producing bacterial strains can be selected for probiotic supplementation. Four weeks of daily *Anaerobutyricum soehngenii* administration dose‐dependently increased fecal concentrations of this butyrate‐producing strain in males with MetS (*n =* 24).^285^ This effect was transient, approximately returning to baseline levels 2‐week postintervention. Despite increased *A*. *soehngenii*, no significant differences in fecal butyrate levels were observed compared with baseline as well as among intervention groups giving a low, medium, or high dose of the probiotic. Peripheral insulin sensitivity, evaluated by hyperinsulinemic‐euglycemic clamp, did not significantly differ between groups, yet peripheral insulin sensitivity was significantly correlated to relative abundance of fecal *A. soehngenii*. This correlation indicates that this bacterial stain may have beneficial effects on insulin sensitivity. However, no correlation was made to the change in (delta) fecal *A. soehngenii*; thus, this observed association is not necessarily related to the intervention itself. Interestingly, exploratory post hoc analysis showed that the ability of *A. soehngenii* intervention to elicit a beneficial glucometabolic response depended on baseline gut microbiota composition. A plausible explanation is that initial bacterial characteristics may influence the engraftment of *A. soehngenii* in the gut microbiome. In another study, patients with T2DM (*n =* 58) were given a mixture of probiotic bacteria along with the prebiotic fiber inulin.^286^ Participants, mainly on antidiabetic medication (metformin), received either placebo WBF‐010 (*Bifidobacterium infantis* and butyrate‐producing *Clostridium Butyricum* and *Clostridium beijerinckii*) or WBF‐011 (containing WBF‐10 plus *Akkermansia muciniphila* and butyrate‐producing *Anaerobutyricum hallii*) for 12 weeks. The latter probiotic mixture improved postprandial glucose response (see Table 1). No effect on fasting glucose, homeostatic model assessment for insulin resistance (HOMA‐IR), or body weight was observed for either of the probiotic mixtures. Strain‐specific qPCR showed fecal that *A*. *halli* was detected more often after 4 and 12 weeks of WBF‐011 supplementation. Unfortunately, fecal *C. Butyricum* and *C*. *beijerinckii* were below detection limit at all time points and it is therefore uncertain whether these bacterial strains were engrafted in the gut microbiome. Intervention‐induced changes in fecal SCFA concentrations were highly variable between participants and not significantly different between groups. Cross‐feeding pathways may partially explain why WBF‐011 mediates stronger metabolic effects. *A*. *muciniphila* may provide acetate which *C. beijerinckii*, *C. butyricum*, and *A*. *hallii* can utilize to form butyrate. Besides this cross‐feeding pathway, *A*. *halli* is also able to convert the lactate produced by *B*. *infantis* to butyrate. Remarkably, participants taking a sulfonylurea agent along with metformin appeared to respond less to WBF‐011 intervention compared with metformin use alone.^286^ These individuals are usually characterized by a longer duration or severity of T2DM. Additionally, metformin is known to modulate the gut microbiome resulting in increased butyrate production and researchers suggest a synergistic relationship between the two.^292^ Hence, one could speculate that WBF‐011 may be more effective in the initial stage of T2DM or that the dose of metformin in these patients was lower, resulting in less synergism. Recently published work revealed that fasting plasma butyrate levels were significantly increased after WBF‐011 intervention compared with placebo, and this was associated with a decrease in HbA_1c_ in individuals that were not using a sulfonylurea agent.^287^ Evidence suggests that some sulfonylurea agents may inhibit the growth of specific bacterial strains present in the WBF‐011 formulation,^287^, ^293^ which may also partially explain the observed reduced treatment outcome in these participants.

Increased fecal and circulating butyrate levels have also been observed in individuals with T1DM after a 6‐week intervention with 40 g of type 2 resistant starch consisting of a high‐amylose (70%) maize starch with acetate and butyrate attached to the dietary fiber.^288^ This increase persisted in week 12, after 6 weeks of follow‐up without intervention, albeit only in the feces and not in the circulation. Although intervention with this modified resistant starch did not alter glucometabolic parameters such as HbA_1c_, circulating butyrate (but not acetate), at week 6, was inversely associated to HbA_1c_, percentage of time that blood glucose concentration was below target range (<3.9 mmol/L), and daily basal insulin requirements. These results suggest that participants who had high butyrate levels at the end of the intervention exhibited better glycaemic control. Next to resistant starch, inulin‐type fructans are also well‐known for their butyrogenic and bifidogenic effects.^69^ Indeed, 3 months of 16 g/day of inulin‐type fructans supplementation increased the abundance of butyrate‐producing *F*. *prausnitzii* in women with obesity compared with participants receiving placebo (maltodextrin).^289^ Although this indicates a butyrate‐inducing effect, the study did not determine actual markers for butyrate production. The glycaemic response after an oral glucose tolerance test was significantly improved compared with placebo, but all other markers of glucometabolic health remained unaffected, except a tendency for inulin‐type fructans to reduce fat mass^289^ (see Table 1). Remarkably, a high dose of fructo‐oligosaccharides (FOS) or galacto‐oligosaccharides (GOS), which are also bifidogenic prebiotics, had detrimental effects on glucose homeostasis in healthy adults.^290^ FOS increased area under the curve for total glucose concentration and GOS significantly increased fasting glucose levels postintervention. Both GOS and FOS supplementation decreased the abundance of several butyrate‐producing bacterial strains, coincided by substantial reduction in fecal butyrate concentrations. Again, considerable heterogeneity in response was identified. Some participants showed improved glycaemic response after GOS intervention yet unfavorable responses after FOS intervention and others vice versa.

### 4.2 Butyrate administration, body weight control, and insulin sensitivity

Instead of elevating colonic microbial butyrate production, butyrate can also be provided orally as an end product itself. Interestingly, 4 weeks of daily oral sodium butyrate supplementation did not affect peripheral nor hepatic insulin sensitivity, measured using the golden standard measurement, in males with MetS (*n =* 10). In contrast, both parameters were significantly increased in healthy males (*n =* 9)^159^ (see Table 2). The provided dose may have been insufficient for individuals with MetS, potentially explaining this discrepancy. After 4 weeks, individuals with MetS exhibit significant reductions in all fecal SCFA, whereas in the plasma, these reductions were limited to propionate only. In line with these results, another clinical trial performed with the same butyrate concentration and intervention duration but including both males and females with MetS (*n =* 12) showed no effects on insulin sensitivity parameters while in this study the fecal SCFA levels remained unchanged. Despite unaltered insulin sensitivity, HbA_1c_ concentration was significantly reduced compared with baseline suggesting that butyrate may mediate minor changes in glucometabolic state.^206^ Nevertheless, the researchers compared the study outcomes of oral butyrate supplementation to a single FMT from a donor that underwent gastric bypass surgery and did not include an additional control group. Two other clinical trials performed with patients with T1DM (45 days of supplementation) and T2DM (4 week of supplementation), respectively, also do not report overt changes in glucose metabolism upon oral sodium butyrate supplementation (see Table 2).^178^, ^283^ Remarkably, butyrate supplementation in patients with T1DM decreased the abundance of butyrate‐producing bacteria and fecal SCFA concentrations.^283^ In patients with T2DM, within‐group analysis revealed the combination of inulin and oral sodium butyrate administration was able to significantly reduce fasting blood sugar and hip‐to‐waist ratio compared with baseline albeit not compared with placebo intervention. Since the use of first‐line medication for T2DM, which could include metformin, prior and during the study was allowed, a synergistic effect with butyrate may have occurred.^178^ Another RCT including patients with T2DM showed that 6 weeks of sodium butyrate supplementation increased fasting plasma insulin compared with baseline albeit not compared with placebo. Remarkably, HOMA‐IR increased significantly compared with initial levels as well as placebo, yet this was no longer significant after adjusting for potential confounding factors including a significant difference in T2DM duration and concentration of antidiabetic medication between the two intervention groups.^239^ Unfortunately, the latter two studies performed with individuals with T2DM did not evaluate a biomarker for butyrate production. None of the above described clinical studies indicate changes in body weight after butyrate intervention, but it remains uncertain whether this may be attributed to the short intervention period (<8 weeks).

**TABLE 2:** Effect of oral butyrate supplementation on weight and glucose metabolic status

Participants	Type + concentration	Design, *duration*, frequency, and timing	Metabolic effects	Study
Healthy lean males (*n =* 9) and males with metabolic syndrome (*n =* 10)	4 g sodium butyrate/day	Clinical trial *4 weeks* Dose divided in 2x/day (no timing specified)	**↑** Peripheral and hepatic insulin sensitivity; but only in lean individuals, not individuals with metabolic syndrome No change in BMI **↓** Fecal total SCFA and fecal butyrate concentration	Bouter et al. (2018)^159^
Adults with metabolic syndrome (*n =* 24)	Autologous fecal transplantation (placebo) and 4 g/day sodium butyrate (*n =* 12) or a single allogenic FMT (from post‐ Roux‐en‐Y gastric bypass donors) and placebo tablets (*n =* 12)	Randomized clinical trial *4 weeks* 1x/day (no timing specified)	Butyrate supplementation **↓** HbA_1c_ compared with baseline No changes in peripheral nor hepatic insulin sensitivity, fasting insulin or fasting glucose No alterations in BMI No changes in fecal SCFA profiles	Hartstra et al. (2020)^206^
Individuals with T1DM (*n =* 30)	4 g sodium butyrate/day or placebo	Cross‐over RCT *4 weeks* Dose divided in 2x/day (no timing specified)	No effect on weight, BMI, residual β‐cell function, HbA_1c_, fasting glucose or daily insulin dose **↓** Abundance of butyrate‐producing strains **↓** Fecal total SCFA and butyrate level	De Groot et al. (2020)^269^
Overweight individuals with T2DM (*n =* 59)	600 mg/day sodium butyrate (*n =* 15), 10 g/day inulin (*n =* 14), combining both sodium butyrate and inulin (*n =* 15), placebo (*n =* 15)	RCT *45 days* Dose divided in 6x/day (after and before each meal)	No changes between intervention groups in BMI, HbA_1c_, HOMA‐IR, fasting blood glucose, and insulin **↓** fasting blood glucose and **↓**waist‐to‐hip ratio for combination intervention compared with baseline but not placebo Did not assess actual biomarkers for butyrate production	Roshanravan et al. (2017)^178^
Overweight individuals with T2DM (*n =* 39)	600 mg/day sodium butyrate (*n =* 20) or placebo (*n =* 19)	RCT *6 weeks* Dose divided in 6x/day (after and before each meal)	No changes in HbA_1c_, fasting blood glucose, and insulin **↑** HOMA‐IR but not after correction for potential confounding variables Did not assess actual biomarkers for butyrate production	Khosravie et al. (2022)^283^

Abbreviations: BMI, body mass index; FMT, fecal microbial transplantation; HbA_1c_, glycated hemoglobin; HOMA‐IR, homeostatic model assessment for insulin resistance; IAUC, incremental area under the curve; RCT, randomized controlled trial; SCFA, short chain fatty acids.

In conclusion, the efficacy of butyrate‐focused human interventions appears modest and is only apparent in within‐group analyses. Nonetheless, the efficacy may depend on the target population and baseline characteristics such as microbiome composition and further investigations are warranted.

## 5 CONCLUDING REMARKS AND FUTURE PERSPECTIVES

Butyrate supplementation studies consistently demonstrate promising beneficial effects on body weight control and insulin sensitivity in animal models. However, whether the experimental design in rodent models is translational to the human situation is questionable. Most of the rodent studies mentioned above provided butyrate in combination with a HFD, before obesity is established, and the time course of development of obesity is not comparable with those in humans. Such an experimental set‐up provides important information about the prevention of obesity but does not give indications on the effect of butyrate when obesity is already present. Moreover, the results of animal studies are not always consistent as some did not find significant alterations in body weight,^204^, ^294^ food/energy intake,^152^, ^153^, ^154^, ^158^, ^182^, ^204^, ^226^ or energy expenditure^209^, ^226^ after butyrate intervention. In humans, Mendelian randomization analysis has inferred a causal relationship between the abundance of several butyrate‐producing microbial strains and an improved postprandial insulin response in normoglycemic individuals.^295^ Nevertheless, so far, human butyrate‐focused intervention studies are scarce and have only demonstrated modest improvements in insulin sensitivity in lean, metabolically healthy, individuals but not in individuals that are metabolically compromised.^159^ The limited available human data are derived from studies with a relatively small sample size and short intervention period (e.g., 4 weeks)^159^, ^206^, ^283^, ^285^, ^290^ and some studies are not placebo‐controlled.^159^, ^206^, ^285^, ^286^, ^288^, ^290^ Additionally, several clinical studies evaluated butyrate status by fecal butyrate concentration, which is not a good proxy for luminal production. The fact that plasma, but not fecal, SCFA levels have been associated with metabolic parameters suggests that plasma SCFA may function as a more adequate biomarker for the metabolic health effects of butyrate.^22^ Besides assessing butyrate levels directly in the circulation, future studies should focus on bacterial activity to study changes in butyrate production and pathways involved in more detail, for example, by using multi‐omics approaches such as metagenomics and metabolomics.^296^ Noninvasive ingestible capsules that enable direct sampling of luminal content may be used to acquire important bioinformation on microbial butyrate production in different regions of the gastrointestinal tract.^297^, ^298^

Numerous studies have reported heterogeneity in the production and kinetics of butyrate after probiotic and prebiotic supplementation,^299^, ^300^, ^301^, ^302^, ^303^, ^304^ which may depend on microbial phenotype and absorption capacity of the host, and this may partially explain interindividual variation in metabolic response towards these interventions. To illustrate, after probiotic intervention, fecal butyrate concentrations were substantially increased in individuals with a low butyrate production at baseline.^301^ However, this increase was significantly less^301^ or even led to reduced fecal butyrate concentration^300^ if initial butyrate levels were already high. These results suggest that initial microbial composition and fecal or plasma butyrate levels could act as a biomarker to preselect individuals that would benefit the most from butyrate‐focused interventions. In addition, the pathological status of the individual, for example, obesity and T2DM as well phenotypic variations and differences in etiology, duration and severity within these pathological states may influence the sensitivity towards butyrate. Consequently, the therapeutic dose of butyrate that is able to elicit beneficial metabolic effects may vary among individuals. Although knowledge on the stability and resilience of the gut microbiome in response to dietary intervention is still largely unknown,^305^ one can hypothesize that the therapeutic window may be more profound at an earlier stage of metabolic dysfunction whereas increased resilience challenges change at a later stage. Nevertheless, the previously reported reduced therapeutic effect of butyrate in individuals and mice with obesity^159^, ^187^ could also be a direct consequence of increased body volume, resulting in a decreased concentration of butyrate per kilogram of fat free mass. Hence, clinical oral butyrate concentrations may need to be changed accordingly (e.g., concentration/kg lean mass), while keeping in mind the preservation of microbial endogenous butyrate production.^82^

Besides the microbial and metabolic phenotype of the participants, other factors that need to be considered are as follows: age, medication use, exercise, sex, stress, genetics, sleep quality, and lifestyle factors including alcohol consumption and smoking. An 8‐week butyrate intervention in obese mice demonstrated a significant reduction in body weight in late‐adult but not mid‐adult mice,^188^, ^229^ suggesting that oral butyrate interventions may be more advantageous at an older age. Since SCFA production and butyrate‐producing bacterial strains appear to be reduced in elderly,^306^ SCFA interventions may be more desired at an older age. Furthermore, several T2DM medications are proposed to have synergistic effects with butyrate including dapagliflozin^157^ and metformin,^292^ suggesting that butyrate has potential to serve as an adjunct to T2DM therapy. Few butyrate‐focused interventions have investigated the effect of sex and ethnicity on study outcomes. Nonetheless, sex and ethnicity‐specific differences in the butyrate producing gut microbiome as well as the response to prebiotic and probiotic interventions have been reported.^307^, ^308^, ^309^, ^310^

Next to interindividual differences, several other components may influence clinical efficacy including intervention duration, concentration and type/form of butyrate or fiber supplied, mode and frequency of administration, and whether butyrate is provided fasted or in the postprandial state (see Table 2). For prebiotic interventions, the level of butyrate production depends, among other factors, on the degree of polymerization and saccharide linkage of the fiber and the intestinal milieu including the abundance of specific microbial strains, for example, *R. bromii*,^49^ the availability of certain B vitamins^311^ and the level and quality of fat.^312^ Recent evidence suggests that different types of dietary fat and the presence of cholesterol (e.g., present in lard but absent in palm oil) may affect gut microbial composition and metabolite profile.^312^, ^313^ This difference may explain why significant reductions in food intake after butyrate intervention have been reported in mice receiving a HFD containing lard as a main dietary fat source^155^, ^177^ but not in mice receiving the same concentration of butyrate but incorporating palm oil as a main dietary fat source.^182^ Other diets that have been associated with a reduced abundance of butyrate‐producing strains or butyrate production include diets high in salt^314^, ^315^ and (animal‐derived) proteins.^316^, ^317^ Lastly, combining exogenous butyrate supplementation with β‐hydroxybutyrate, a ketone body, may induce synergistic metabolic effects for weight loss.^318^ Overall, food‐microbe crosstalk may explain inconsistencies among animal studies and may interact with the outcome of human butyrate interventions. To optimize butyrate‐focused prebiotic interventions, substrate supply and initial presence of specific bacterial communities need to be considered.

Next to the dose of butyrate and dietary context, the level of butyrate that reaches the circulation may depend on where butyrate is absorbed along the gastrointestinal tract. In the colon, butyrate maintains energy homeostasis as a result of a mutualistic relationship between host and butyrate‐producing microbes.^100^, ^101^, ^144^, ^145^ However, in the upper part of the intestine, microbes (including butyrate‐producing bacteria) are present in sustainably lower amounts.^69^ Since enterocytes prefer other energy sources such as glucose over butyrate, oral butyrate supplementation may increase the amount of butyrate reaching the liver and circulation compared with colonically derived butyrate. Interestingly, butyrate can partly bypass the liver via the internal iliac vein in the distal part of the colon.^319^ Supplementing dietary fibers that ferment more distally or administering butyrate enemas in the rectum could potentially increase circulating butyrate levels. A study investigating acetate administration along the gastrointestinal tract in men with obesity already demonstrated profound beneficial metabolic alterations after distal but not proximal administration.^320^ Whether such differences also exist for butyrate still needs to be investigated. Yet, a recent study demonstrated that combining long‐chain inulin with resistant starch increased fasting plasma butyrate, coincided by beneficial metabolic effects including an increased energy expenditure, compared with inulin alone in healthy men.^321^ This fiber combination may potentially reach the colon more distally, explaining the observed increased systemic butyrate levels.

Overall, solid statements about the potential metabolic benefits of butyrate‐focused interventions in humans remain premature and are likely highly context specific. In order to tilt microbial disturbances and impaired metabolic processes, interventions may require a personalized approach and a longer intervention period. Future studies should specify whether the optimal dose of butyrate differs for specific target populations, for example, individuals with obesity and individuals using metformin and elucidate the optimal mode, frequency, and (dietary) context of butyrate intake. Lastly, the controversy on the role of butyrate in individuals with metabolic disturbances needs to be disentangled. Future research should elucidate whether butyrate is an important etiological factor in the prevention and management of obesity and obesity‐related complication and determine which processes in carbohydrate fermentation and SCFA handling are altered. Whether obesity and T2DM dysregulates butyrate production, absorption, clearance, and/or alters the sensitivity towards butyrate provides crucial information that can be fundamental for improving the efficacy of butyrate‐focused clinical trials.

## CONFLICT OF INTEREST

The authors declare no conflict of interest.
